# A biomimetic nano-NET strategy for the treatment of MRSA-related implant-associated infection†

**DOI:** 10.1039/d5ra00367a

**Published:** 2025-05-07

**Authors:** Huan Xu, Dengwei He, Huimin Tao

**Affiliations:** a Department of Orthopedics Surgery, Lishui Hospital, Zhejiang University School of Medicine No. 289, Kuocang Road Lishui Zhejiang 323000 China hedw_spine@163.com; b Department of Orthopedics Surgery, The Second Affiliated Hospital, Zhejiang University School of Medicine No. 88, Jiefang Road Hangzhou Zhejiang 310009 China huimintao_zrgk@163.com

## Abstract

Methicillin-resistant *Staphylococcus aureus* (MRSA) has spread across diverse global environments, and MRSA-related infection is a major threat to public health. Implant-associated infection (IAI) caused by MRSA remains a tough global clinical problem. Conventional antibiotic therapy has limited efficacy in treating MRSA-related IAI, and antibiotic abuse has resulted in the emergence of multidrug-resistant bacteria. Hence, there is a necessity to explore more effective approaches to deal with MRSA-related IAI. Herein, inspired by neutrophil extracellular traps (NETs) released by neutrophils to kill microorganisms, this study proposes a novel biomimetic nano-NET strategy using an epsilon-poly-l-lysine-coated CuO_2_ nanoplatform, denoted as PCPNAs. The function-adaptive nanoplatform exhibited excellent Fenton-like performance, including robust ROS generation and GSH scavenging ability. PCPNAs showed >90% cell viability in mammalian cells and reduced bacterial burden by 7.65 log_10_ CFU *in vitro*. Moreover, the positively charged PCPNAs could easily bind to negatively charged MRSA cells through charge-coupling and simultaneously exerted a trapping effect on MRSA cells. Notably, PCPNAs self-assembled into web-like structures to physically trap and kill biofilm bacteria, achieving 99.58% biofilm eradication. Furthermore, PCPNAs showed satisfactory biocompatibility *in vivo* and displayed ideal anti-bacterial and anti-inflammatory effects in a mouse model with implant-associated infection. With further development and optimization, the biomimetic nano-NET strategy based on PCPNAs provides a new therapeutic option for the treatment of MRSA-related implant-associated infection.

## Abstract 2

Schematic of a biomimetic nano-NET strategy based on PCPNAs for the treatment of MRSA-related implant-associated infections.

## Graphical abstract

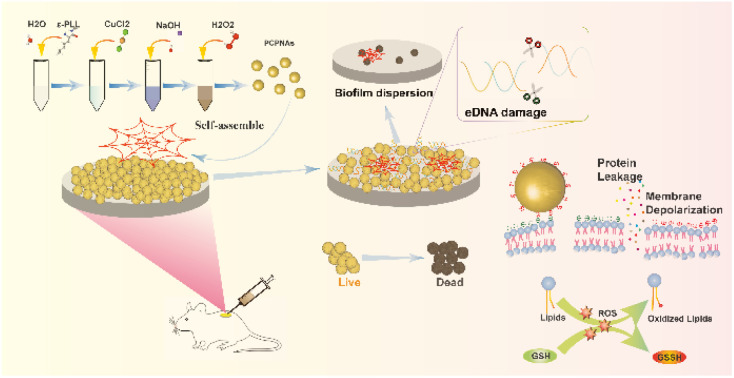

## 1. Introduction

Biomedical implants have been widely used in clinical diagnosis and treatment. However, the ensuing implant-associated infection (IAI) is one of their most frequent and catastrophic complications, causing prolonged antibiotic therapy or secondary surgery.^1,2^ The incidence of infective endocarditis after left-sided heart valve replacement is 0.894%.^3^ The rate of periprosthetic joint infection after secondary procedure for periprosthetic fractures is as high as 6.8% to 11.1%.^4^ Device-associated infections account for the largest proportion (25.6%) of all health care-related infections in the United States.^5^ IAI is peculiarly difficult to deal with and becomes an enormous financial burden for global healthcare systems.^2,6^ Bacteria play a vital role in the occurrence of IAI. Methicillin-resistant *Staphylococcus aureus* (MRSA), which is a major cause of hospital-acquired infections, is one of the most common pathogenic bacteria for IAI.^7–9^ MRSA can adhere to the surface of implants to form biofilms, which can shelter bacteria and cause refractory infection.^1^ Notably, MRSA harbors critical antibiotic resistance genes, such as tetK, ermC, blaZ and dfrA, and is resistant to nearly all conventional antibiotics.^8^ MRSA infection is a severe threat to global public health.^10^ Currently, antibiotics such as vancomycin remain the main method for the treatment of IAI.^2,11^ Unfortunately, antibiotic abuse has led to the emergence of multidrug-resistant bacteria.^12^ For example, vancomycin has been used clinically in MRSA infection, but now vancomycin-resistant *Staphylococcus aureus* is prevalent worldwide.^13^ Moreover, antibiotic therapies can lead to a range of serious side effects, such as hepatotoxicity and Stevens–Johnson syndrome.^14,15^ Thus, to address the challenge of IAI, it is necessary to develop novel drug-free antimicrobial therapies.

The emerging technique of nanotechnology has provided potential solutions for the treatment of bacterial infections and IAI.^16–21^ Currently, Fenton/Fenton-like reaction-based chemodynamic therapy has become a mainstream non-antibiotic therapeutic strategy for IAI.^22^ Metal-based nanomaterials, such as ferrosoferric oxide (Fe_3_O_4_) and copper peroxide (CuO_2_), are excellent Fenton/Fenton-like reagents, which can catalyze hydrogen peroxide (H_2_O_2_) to form hydroxyl radicals (˙OH), the most toxic reactive oxygen species (ROS).^23,24^ The generation of ˙OH *via* Fenton/Fenton-like reaction relies on the supply of H_2_O_2_. However, bacterial biofilms contain a low level of H_2_O_2_, which limits the antibacterial effect of chemodynamic therapy without exogenous H_2_O_2_.^25,26^ CuO_2_ nanoparticles (NPs) can react with hydrogen ion under acidic conditions to self-supply H_2_O_2_ and copper ion (Cu^2+^), a robust Fenton catalyst.^27^ Several studies have investigated the antibacterial activity of CuO_2_ NPs.^28,29^ For example, Wang's group synthesized dextran-coated CuO_2_ NPs, which displayed acid-induced ROS generation and anti-biofilm ability.^28^ Zhu's group constructed mesoporous silica nanoshell-encapsulated CuO_2_ NPs; the NPs exhibit Fenton catalytic activity and antibacterial effects on infected wounds.^29^ However, CuO_2_ NPs designed for antimicrobial applications fail to establish effective interactions with bacteria, which would diminish the antimicrobial activity because of the ultrashort lifetime (<200 nanoseconds) and diffusion length (roughly 20 nanometers) of ROS.^30,31^ Moreover, uncontrolled ROS will be toxic to normal cells while killing bacteria.^32,33^ Therefore, it is imperative to develop novel strategies to address these challenges facing CuO_2_ NPs. Recently, positively charged cationic polymers that can interact with negatively charged bacterial membrane by electrostatic binding have been selected as potential candidates for antibacterial therapy.^34,35^ Among various surface modification strategies, the selection of epsilon-poly-l-lysine (ε-poly-l-lysine) (ε-PLL) as a coating material for CuO_2_ NPs is driven by its unique physicochemical properties and biological origin. ε-PLL is a type of natural cationic polymer containing abundant l-lysine residues.^36^ As a U.S. FDA-designated generally recognized as safe substance, ε-PLL possesses excellent biological adhesive capacity, water solubility and biocompatibility and has been extensively utilized in food and medical fields, such as drug delivery and antibacterial treatment.^37^ We envisioned that combining CuO_2_ NPs with ε-poly-l-lysine would provide a new method to address the aforementioned shortcomings of CuO_2_ NPs and treat the MRSA-related IAI. As a microbially derived cationic polypeptide, the cationic biopolymer ε-PLL can mimic LL-37 in neutrophil granules – the only human cathelicidin that utilizes cationic domains (containing 6 Lys residues) to target anionic prokaryotic membranes with charge-selective precision.^38^ The rationale for integrating ε-PLL with CuO_2_ NPs is driven by two synergistic mechanisms: first, cationic residues in ε-PLL can selectively bind to anionic bacterial membranes *via* coulombic attraction,^39^ enabling pathogen-specific nanoparticle accumulation at infected sites and ensuring a localized ROS storm. Second, ε-PLL may mimic the natural interplay between host-derived polymers (such as neutrophil granule-derived LL-37) and pathogens and help nanoparticle entrapping of bacteria. Consequently, the ε-PLL-CuO_2_ NPs may achieve synergistic integration of MRSA immobilization and microenvironment-triggered ROS generation localized to acid infection sites through a two-pronged antibacterial mechanism unattainable by traditional monofunctional synthetic agents.

**Scheme 1:**
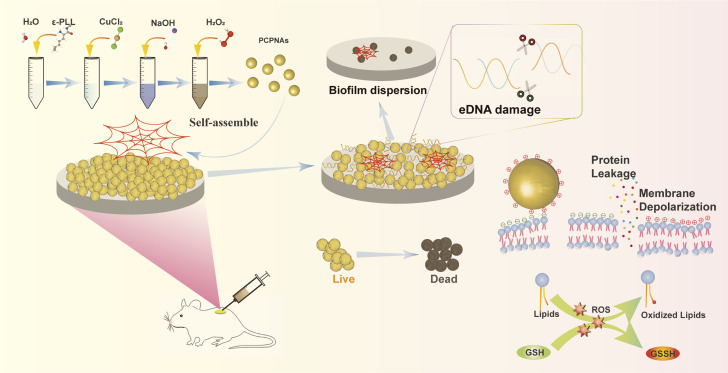
Schematic of biomimetic nano-NET strategy based on PCPNAs for the treatment of MRSA-related implant-associated infection.

Herein, inspired by neutrophil extracellular traps (NETs) released by neutrophils to kill microorganisms, we propose a novel biomimetic nano-NET strategy based on a nanoplatform to deal with MRSA-related IAI (Scheme 1). The nanoplatform was synthesized using a simple one-pot strategy. CuO_2_ is coated with ε-PLL to form NPs (hereafter called PCPNAs). ε-PLL provides a cationic character for PCPNAs, which can respond to negative charges and guide PCPNAs to anchor on the surface of bacterial cell envelopes. CuO_2_ can decompose to Cu^2+^ and H_2_O_2_ and subsequently trigger a Fenton-like reaction to explosively release toxic ˙OH. Simultaneously, the released Cu^2+^ can further scavenge glutathione (GSH) inside biofilms to enhance antibacterial effect of ˙OH. More interestingly, PCPNAs could self-agglomerate together to form web-like structures to physically trap and kill bacteria like NETs. The novel nanoplatform exhibited excellent antibacterial activity *in vitro*, and a mouse model of implant-related infection was established to further investigate its antibacterial effect.

## 2. Materials and methods

### 2.1. Materials

Sodium hydroxide (NaOH) was purchased from Xilong Scientific Co., Ltd. Hydrogen peroxide (H_2_O_2_, 30%) was purchased from Shanghai Lingfeng Chemical Reagents Co., Ltd. Copper(ii) chloride hexahydrate (CuCl_2_·2H_2_O) and ε-polylysine hydrochloride (ε-PLL) were purchased from Shanghai Aladdin Biochemical Technology Co., Ltd. 3,3′,5,5′-Tetramethylbenzidine (TMB), 3,3′-dipropylthiadicarbocyanine iodide (DiSC_3_5), 5,5′-dithiobis-(2-nitrobenzoic acid) (DTNB) and lysozyme (40 000 U mg^−1^) were purchased from Shanghai Macklin Biochemical Co., Ltd. Luria–Bertani (LB) broth powder and LB agar powder were purchased from Sangon Biotech (Shanghai) Co., Ltd. Bradford protein assay kit, InstantView™ red fluorescent DNA Loading buffer (6× with BeyoRed), InstantView™ red fluorescent DNA ladder (0.2–12 kbp, 12 bands, bromophenol blue) and 2′,7′-dichlorodihydrofluorescein (DCFH-DA) were purchased from Beyotime Biotechnology, Shanghai, China. Tris acetate–EDTA buffer (TAE, 50×) were purchased from Biosharp Co., Ltd (Beijing, China). Agarose was purchased from Baygene Biotechnologies Co., Ltd (Shanghai, China). C11-BODIPY^581/591^ and a LIVE/DEAD BacLight bacterial viability kit were purchased from Thermo Fisher Scientific Inc. Cell Counting Kit-8 (CCK-8) was purchased from APExBIO Technology LLC. All reagents in this study were used without any further purification.

### 2.2. Synthesis of PCPNAs

First, 190 mg of ε-PLL was added into a 50 mL centrifuge tube and ultrasonically dissolved in 20 mL of deionized water. Subsequently, 1 mL of CuCl_2_·2H_2_O (0.5 M) was administered in the above solution and magnetically stirred at 1000 rpm for 2 min. Afterward, 1 mL of NaOH (1 M) was pipetted in the above mixed solution and continuously stirred for 10 min. Then, 1 mL of H_2_O_2_ (30%) was added, followed by stirring in an ice bath for 50 min to obtain a dark brown clear solution. Finally, the PCPNAs were ultrafiltrated at 3000×*g* (Avanti J-15R, rotor JS-4.750, radius: 20.78 cm) using PALL centrifugal devices (10 kDa) and washed with deionized water three times. The product was freeze-dried and stored at −20 °C for further use.

### 2.3. Characterizations

Transmission electron microscope (TEM) observations were performed with a JEM-1230 electron microscope (Japan). Field emission scanning electron microscopes (FE-SEM) images were obtained on an ultra-high resolution microscope (SU8010, Hitachi, Japan). The particle size and zeta potentials were measured by a Malvern ZEN 3600 zetasizer (UK). X-ray photoelectron spectroscopy (XPS) for detecting surface elements were performed using a Thermo Scientific K-Alpha instrument (USA). The content of copper ions in bacteria was detected by inductively coupled plasma-mass spectrometry (ICP-MS, Agilent 7800). ^1^H-NMR spectra were taken using a Bruker 600 MHz NMR spectrometer in deuterium chloride solution. Fourier transform infrared (FT-IR) analysis of PCPNAs was conducted using the KBr pellet method with a resolution of 4 cm^−1^ and 20 scans from 4000 to 400 cm^−1^ (IRSpirit, Japan). UV absorption spectra of PCPNAs dissolved in an equal-volume mixture of PBS (pH 7.4) and hydrochloric acid (0.1 M) were measured using a UV-Vis spectrophotometer (UV-1900i, Japan). The coating efficiency and stability of ε-PLL in PCPNAs were also measured using a UV-Vis spectrophotometer.

### 2.4. pH-dependent ˙OH generation assay

10 μL of TMB solution (4 mg mL^−1^) was added to 1 mL of PCPNAs (100 μg mL^−1^), ε-PLL (100 μg mL^−1^), and PBS as control at different pH values (5.0 and 7.4), separately. 100 μL aliquots of the above suspensions were pipetted into a 96-well plate. After mixing for 60 min, the absorbance was measured at 650 nm using a Spark multimode microplate reader (Tecan, Switzerland).

### 2.5. GSH depletion assay

DTNB (40 mg mL^−1^) was added to bicarbonate buffer (0.05 M, pH = 8.7). Tris–HCl (0.05 M, pH = 8.0) and the above solution were mixed in a 40 : 1 ratio to form DTNB-bicarbonate-buffer–Tris–HCl buffer. PCPNAs (100 μg mL^−1^), ε-PLL (100 μg mL^−1^), and 30% H_2_O_2_ (10 μL mL^−1^, positive control) were individually introduced into a GSH solution (400 μg mL^−1^ in PBS, pH 5.0), along with PBS as the negative control. Subsequently, 400 μL of the resulting solutions were mixed with 2 mL of DTNB-bicarbonate–Tris–HCl buffer. After 6 h of incubation, 100 μL aliquots of the above suspensions were pipetted into a 96-well plate. The absorbance was recorded at 412 nm on a Spark multimode microplate reader (Tecan, Switzerland). The clearance rate of GSH was calculated as follows (eqn (1)):1
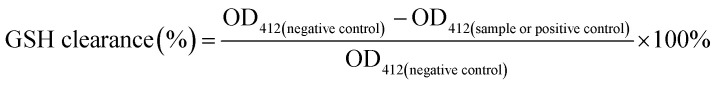


### 2.6. Cytotoxicity assay

The cytotoxicity of PCPNAs was assessed using a CCK-8 test with a C2C12 (National Collection of Authenticated Cell Cultures) cell line cultured in Dulbecco's Modified Eagle's Medium (DMEM) supplemented with 10% fetal bovine serum (FBS) at 37 °C under a 5% CO_2_ atmosphere. In brief, the cells (2 × 10^4^ per well) were seeded in a 96-well plate and cultured for 24 h before removing the supernatant. PCPNAs were dispersed in DMEM (10% FBS) and filtered through a 0.22 μm syringe filter twice for sterilization. Subsequently, the cells were incubated with PCPNAs at various concentrations for another 24 h. The media were removed, and cells were washed once with PBS. Then, 100 μL of work solution (CCK-8 reagent: DMEM (10% FBS) = 1 : 10, v/v) was pipetted into each well, followed by further incubation for 2 h. The absorbance of each well at 450 nm was detected with a Spark multimode microplate reader (Tecan, Switzerland).

### 2.7. Bacterial culture and plate counting assay

Methicillin-resistant *Staphylococcus aureus* (MRSA) (ATCC 43300) and MRSA USA300-GFP (Beijing Beina Chuanglian Biotechnology Institute) were selected as model bacteria. Bacteria was inoculated into 5 mL of LB liquid medium and incubated in an orbital shaker at 37 °C and 120 rpm for 6 h. The bacteria suspension was centrifuged at 5000×*g* for 5 min at 4 °C (Thermo Scientific Sorvall ST 16R, rotor F15-6 × 100 y, radius: 9.8 cm) and resuspended in 1 mL of PBS. Afterwards, 100 μL aliquots of the bacterial suspension were pipetted into a 96-well plate and the OD_600_ values were detected using a Spark multimode microplate reader (Tecan, Switzerland). The bacterial suspension was then gradually diluted to 10^−7^ folds. Subsequently, 100 μL of diluted suspension was plated on a LB agar plate and then incubated at 37 °C for 24 h. The colony-forming units (CFUs) on the LB agar plate were counted and photographed with a camera.

### 2.8. Zeta potential measurement of MRSA

MRSA was cultured as above and resuspended in normal saline to 5 × 10^9^ CFU mL^−1^. Briefly, 1 mL of MRSA suspension in normal saline (5 × 10^9^ CFU mL^−1^) was pipetted into 8 mL of normal saline and then mixed with 1 mL PCPNAs (1 mg mL^−1^), ε-PLL (1 mg mL^−1^) and normal saline as a control. The mixed solutions were cultured at 37 °C and 120 rpm for 1 h, centrifuged at 5000×*g* for 5 min at 4 °C (Thermo Scientific Sorvall ST 16R, rotor F15-6 × 100 y, radius: 9.8 cm) and washed three times with normal saline. The pellets were resuspended in 2 mL of normal saline. The zeta potential of the resuspended MRSA was detected using a Malvern ZEN 3600 zetasizer (UK).

### 2.9. Adhesion capacity of PCPNAs

MRSA USA300-GFP was dispersed in normal saline with OD_600_ = 1.3. Two hundred μL of bacterial suspension was pipetted into 2 mL of PCPNAs (200 μg mL^−1^), ε-PLL (200 μg mL^−1^) and normal saline as a control. The initial OD_600_ (OD_0min_) of the above suspensions were recorded with a multifunctional microplate reader (Infinite M200 Pro, Tecan, Switzerland). After 10 min of incubation, the OD_600_ of the supernatant of the previous solutions were recorded as OD_15min_. The relative OD_600_ (%) was calculated as follows (eqn (2)):2
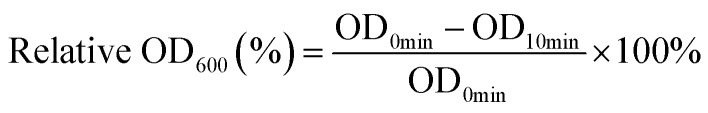


The residual solutions were centrifuged at 3000×*g* for 3 min (Eppendorf 5424R, rotor FA-45-24-11, radius: 8.4 cm) and washed twice with normal saline. The pellets were dispersed in 1 mL normal saline. A 5 μL aliquot of the bacterial suspension was pipetted onto a slide and covered with a coverslip. The morphology of the bacteria was observed using a fluorescence microscopy (Leica, Germany).

### 2.10. ICP-MS of bacterial copper

Briefly, 1 mL MRSA suspension in normal saline (OD_600_ = 0.6) was pipetted into 8 mL normal saline and then mixed with 1 mL PCPNAs (1 mg mL^−1^), ε-PLL (1 mg mL^−1^) and normal saline as a control. The mixed solutions were cultured at 37 °C and 120 rpm for 12 h. After incubation, the mixtures were centrifuged at 5000×*g* for 5 min (Thermo Scientific Sorvall ST 16R, rotor F15-6 × 100 y, radius: 9.8 cm), washed thrice with deionized water and then lyophilized in a 15 mL EP tube. Afterwards, the pellets were digested at 100 °C for 30 min using 200 μL of 65% nitric acid solution. 2.8 mL of deionized water was pipetted into each EP tube. The lysate in each tube was filtered through a 0.22 μm syringe filter into a 5 mL EP tube and then analyzed using ICP-MS (7800, Agilent).

### 2.11. Cytoplasmic membrane depolarization assay

Briefly, 0.1 mL of MRSA suspension (OD_600_ = 1.0), 1 μL of DiSC_3_5 (5 mM) and 0.8 mL of deionized water were pipetted into a 24-well plate and incubated in the dark at 37 °C and 120 rpm for 30 min. Subsequently, 0.1 mL PCPNAs (1 mg mL^−1^), ε-PLL (1 mg mL^−1^) and deionized water (control) were added into the mixed suspension and incubated for another 2 h. Two hundred μL aliquots of the above suspensions were placed in a 96-well plate. Changes in fluorescence intensity were recorded (excitation *λ* = 510 nm, emission *λ* = 670 nm) with a Spark multimode microplate reader (Tecan, Switzerland).

### 2.12. Evaluation of bacterial cell leakage

The leakage of bacterial protein was measured using Coomassie brilliant blue G-250 of the Bradford protein assay kit. Briefly, 0.2 mL of MRSA suspension in normal saline (OD_600_ = 1.6) was applied to 1.7 mL of normal saline and then mixed with 0.1 mL PCPNAs (4 mg mL^−1^), ε-PLL (4 mg mL^−1^) and normal saline as a control. The mixtures were cultured at 37 °C and 200 rpm for 12 h. The supernatant was collected by centrifugation at 7000×*g* for 5 min at 4 °C (Eppendorf 5424R, rotor FA-45-24-11, radius: 8.4 cm) and then mixed with the Coomassie brilliant blue G-250 solution in a 1 : 2 ratio. Normal saline and the Coomassie brilliant blue G-250 solution were mixed in a 1 : 2 ratio as the blank group. Afterwards, 200 μL aliquots of the above solutions were pipetted into a 96-well plate and the OD_595_ values were detected using a multifunctional microplate reader (Infinite M200 Pro, Tecan, Switzerland). The ratio of bacterial protein leakage was calculated as follows (eqn (3)):3
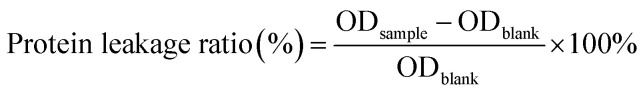


### 2.13. DNA damage assay

Two microliters of MRSA suspension in PBS (OD_600_ = 0.3) was centrifuged at 10 000×*g* for 5 min (Eppendorf 5424R, rotor FA-45-24-11, radius: 8.4 cm). The bacterial pellet was lysed with 200 μL of lysozyme at 37 °C for 120 min. The genomic DNA (gDNA) of the lysate was extracted using a FastPure® bacteria DNA isolation mini kit (Vazyme, China). Subsequently, 50 μL of DNA solution was mixed with 50 μL PCPNAs (0.2 mg mL^−1^), ε-PLL (0.2 mg mL^−1^) and deionized water as a control. The mixtures were incubated at 37 °C and 120 rpm for 6 h. The above solutions were mixed with InstantView™ red fluorescent DNA loading buffer in a 5 : 1 ratio. The gDNA cleavage products were analyzed by electrophoresis using a 1% agarose gel in TAE buffer. DNA bands were visualized using a gel imaging system (Universal Hood II, Bio-Rad, USA).

### 2.14. Intracellular ROS assay

DCFH-DA dissolved in normal saline (10 μM) was used to detect the intracellular ROS triggered by various treatments in MRSA cells. Fresh MRSA cells were dispersed in deionized water to give a concentration of 1 × 10^9^ CFU mL^−1^ and then incubated with PCPNAs (100 μg mL^−1^), ε-PLL (100 μg mL^−1^) and normal saline as a control at 37 °C and 120 rpm for 2 h. After incubation, the mixtures were centrifuged at 10 000×*g* for 5 min at 4 °C (Eppendorf 5424R, rotor FA-45-24-11, radius: 8.4 cm) and washed twice with 1 mL of normal saline. The bacterial pellets were added to 0.4 mL of DCFH-DA solution, incubated for another 30 min, and then washed with 1 mL of normal saline for three times. The pellets were resuspended with 1 mL of normal saline. Afterwards, 200 μL aliquots of the above suspensions were pipetted into a 96-well black plate. The fluorescence intensity was recorded (excitation *λ* = 485 nm, emission *λ* = 535 nm) with a Spark multimode microplate reader (Tecan, Switzerland). Five μL aliquots of the bacterial suspensions were pipetted onto a slide, covered with a coverslip and observed using fluorescence microscopy (Leica, Germany) (excitation *λ* = 450–490 nm, emission *λ* = 515 nm).

### 2.15. Intracellular lipid peroxide (LPO) measurement

A C11-BODIPY^581/591^ probe dispersed in normal saline (10 μM) was used to detect the intracellular LPO levels generated by different treatments in MRSA cells. Briefly, 1 mL of MRSA suspension in normal saline (OD_600_ = 0.9) was centrifuged at 5000×*g* for 5 min (Eppendorf 5424R, rotor FA-45-24-11, radius: 8.4 cm) and then co-incubated with 1.5 mL of C11-BODIPY^581/591^ probe at 37 °C and 120 rpm for 1 h. The mixture was centrifuged, washed twice with normal saline and then redispersed in 1 mL of normal saline. MRSA cells were loaded with C11-BODIPY^581/591^ probe. Subsequently, 1 mL of MRSA suspension was applied into 8 mL of normal saline and then incubated with PCPNAs (1000 μg mL^−1^), ε-PLL (1000 μg mL^−1^) and normal saline as a control at 37 °C and 120 rpm for 2 h. After incubation, the mixtures were centrifuged at 5000×*g* for 5 min at 4 °C (Eppendorf 5424R, rotor FA-45-24-11, radius: 8.4 cm), and washed twice with normal saline. The bacterial pellets were resuspended with 1 mL of normal saline. Afterwards, a 5 μL aliquot of the bacterial suspension was pipetted onto a slide, covered with a coverslip and observed using fluorescence microscopy (Leica, Germany) (excitation *λ* = 517–563 nm, emission *λ* = 590 nm). Two hundred μL aliquots of the above suspensions were analyzed by flow cytometry (CytoFLEX LX, Beckman).

### 2.16. Real-time quantitative reverse transcription PCR (qRT-PCR) analysis

MRSA cells were first incubated with PCPNAs (100 μg mL^−1^), ε-PLL (100 μg mL^−1^) and normal saline as a control at 37 °C for 10 h. After incubation, the mixtures were centrifuged and washed thrice with normal saline. Total RNA of MRSA was extracted using a bacteria RNA extraction kit (Vazyme, China) and then converted to complementary DNA (cDNA) using Hifair® III 1st Strand cDNA Synthesis SuperMix (Yeasen, China) according to the manufacturer's protocol. The expression of the *icaR* gene was assessed by qRT-PCR using Hieff UNICON® qPCR SYBR Green master mix (Yeasen, China) and an Applied Biosystems 7500 Fast real-time PCR system (Thermo Fisher Scientific) in a twenty microliters of reaction volume per well. The 16S rRNA gene was used as the internal reference. The forward and reverse primers (5′ to 3′) of the *icaR* gene were TGCTTTCAAATACCAACTTTCAAGA and ACGTTCAATTATCTAATACGCCTGA, respectively. The forward and reverse primers (5′ to 3′) of the 16S rRNA gene were GGGACCCGCACAAGCGGTGG and GGGTTGCGCTCGTTGCGGGA, respectively. The 2(-Delta Delta C (T)) method was used to quantify the expression level of the icaR gene.^40^

### 2.17. Live/dead staining assay

Live/dead staining assay was performed using the LIVE/DEAD BacLight bacterial viability kit (Thermo Fisher Scientific Inc.). A 1 : 1 mixture of SYTO 9 dye and propidium iodide (PI) was diluted 1000-fold with PBS. One microliter of MRSA suspension in normal saline (OD_600_ = 1.2) was added to 8 mL of normal saline and then incubated with 1 mL PCPNAs (1000 μg mL^−1^), ε-PLL (1000 μg mL^−1^) and normal saline as a control at 37 °C and 120 rpm for 9 h. After incubation, the mixtures were centrifuged at 5000×*g* for 5 min at 4 °C (Thermo Scientific Sorvall ST 16R, rotor F15-6 × 100 y, radius: 9.8 cm), and washed three times with PBS. The bacterial pellets were resuspended with 1 mL of dye solution and incubated at 37 °C and 120 rpm for 1 h. Afterwards, the bacterial suspension was centrifuged at 5000×*g* for 5 min at 4 °C (Thermo Scientific Sorvall ST 16R, rotor F15-6 × 100 y, radius: 9.8 cm), and washed three times with PBS. The pellets were resuspended with 2 mL of PBS. Afterwards, 10 μL aliquots of the bacterial suspension were pipetted onto a slide, covered with a coverslip and observed using fluorescence microscopy (Leica, Germany) (green integrated fluorescence: excitation *λ* =450–490 nm, emission *λ* = 515 nm; red integrated fluorescence: excitation *λ* = 517–563 nm, emission *λ* = 590 nm). The integrated fluorescence density of each image was calculated using ImageJ software (1.54d). The death rate of the samples was calculated as follows (eqn (4)):4
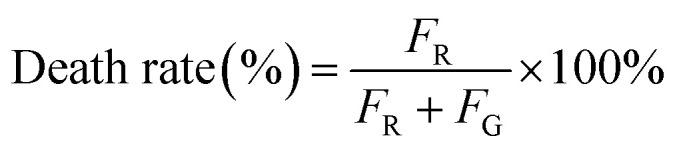
*F*_R_ is the red integrated fluorescence density of the samples, while *F*_G_ is the green integrated fluorescence density.

### 2.18. Observation of bacterial morphology

Briefly, MRSA (OD_600_ = 0.1) treated with PCPNAs, ε-PLL and normal saline was fixed with 2.5% glutaraldehyde at 4 °C for 60 min. The samples were centrifuged at 5000×*g* (Thermo Scientific Sorvall ST 16R, rotor F15-6 × 100 y, radius: 9.8 cm) and washed with PBS for three times, followed by an ethanol dehydration at a series of concentrations (30%, 50%, 70%, 80%, 90%, 95% and 100%). The bacterial precipitate was resuspended in absolute alcohol, pipetted on a silicon wafer and coated with platinum by sputtering. Bacterial morphology was observed by FE-SEM (SU8010, Hitachi, Japan).

### 2.19. *In vitro* antibacterial assay

A 10 μL aliquot of MRSA suspension (5 × 10^9^ CFU mL^−1^) was incubated in 990 μL of normal saline with PCPNAs (100 μg mL^−1^), ε-PLL (100 μg mL^−1^) or normal saline as a control at 37 °C and 120 rpm for 3.5 h, followed by dilution to 10 000 times. Subsequently, 100 μL of diluted suspension was plated on an LB agar plate and then incubated at 37 °C for another 24 h. The number of CFUs in each group was counted and photographed with a camera.

The antibacterial effect of PCPNAs was further studied using the minimal inhibitory concentration (MIC) and time-kill kinetics assay. To evaluate the MIC of PCPNAs, serial twofold dilutions of PCPNAs (25–200 μg mL^−1^) were prepared in sterile EP tubes, followed by inoculation with MRSA suspensions standardized to 2 × 10^6^ CFU mL^−1^ in LB solutions for 24 h at 37 °C. Untreated MSRA was used as a negative control. The MIC value was determined as the lowest PCPNA concentration that inhibits MRSA growth when the treated bacterial counts (in CFU ml^−1^) were lower than the initial bacterial counts.

Also, the time-kill kinetic assay of PCPNAs was evaluated quantitatively. MRSA suspensions (2 × 10^6^ CFU mL^−1^ in LB solution) were exposed to PCPNAs at 2× the MIC concentration. Untreated MSRA served as a negative control. Aliquots (100 μL) were aseptically collected at optimized intervals (0, 1, 2, 4, 8, and 24 h), serially diluted in sterile LB, and plated on LB agar for colony enumeration.

### 2.20. Biofilm dispersion assays

Crystal violet staining was applied to evaluate the ability of PCPNAs to disperse a biofilm. Sterile silicon pieces (diameter, 6 mm) were placed in a 24-well plate. Two microliters of MRSA suspension (1 × 10^7^ CFU mL^−1^) in LB was added to each well and cultured at 37 °C for 72 h to form a biofilm on the surface of silicon pieces. The biofilm-loaded silicon pieces were rinsed with deionized water to remove planktonic bacteria and then transferred to a new 24-well plate, followed by treatment with 2 mL PCPNAs (100 μg mL^−1^), ε-PLL (100 μg mL^−1^) or normal saline as a control. After incubation for 2.5 h at 37 °C, the silicon pieces were rinsed with deionized water and air-dried. The biofilm was fixed with 10 μL of methanol for 15 min. After drying in air, the biofilm was dyed with 30 μL of crystal violet (0.5%, w/v) for 15 min, followed by rinsing with deionized water to remove excess dye and air-dried. The morphology of the biofilm was photographed with a camera. Subsequently, 0.5 mL of acetic acid solution (33%, v/v) was applied into each well to release dye combined in the biofilm. Then, 100 μL aliquots of the dissolved dye solution were pipetted into a 96-well plate. The absorbance was recorded with a Spark multimode microplate reader (Tecan, Switzerland) at 590 nm.

Biofilm dispersion by PCPNAs was also observed by three-dimensional (3D) CLMS. Biofilms cultured on culture dishes were treated with 2 mL of PCPNAs (200 μg mL^−1^), ε-PLL (200 μg mL^−1^), or normal saline as a control. After incubation for 5 h at 37 °C, the culture dishes were rinsed with normal saline and stained with a mixed solution of Calcein-AM (2 μM) and PI (10 μM) for 30 min at 37 °C in the dark. The biofilms were rinsed with normal saline, covered with anti-fluorescence quenching agent and the 3D biofilm fluorescence spots were reconstructed by CLSM (Leica DMi8, Germany).

The morphology of biofilm treated with PCPNAs was also investigated through FE-SEM. The biofilm on a glass coverslip was treated with PCPNAs, ε-PLL or normal saline as a control and was fixed with glutaraldehyde (2.5%) overnight at 4 °C. After rinsing with PBS (0.1 M) thrice, the biofilm was dehydrated with ethanol at a series of concentrations (30%, 50%, 70%, 80%, 90%, 95% and 100%) and sputtered with platinum. The morphology of the biofilm was observed with FE-SEM (SU8010, Hitachi, Japan).

### 2.21. PCPNAs in the treatment of implant-related infection

The animal experiment was approved by the Animal Ethics Committee of the Second Affiliated Hospital, Zhejiang University School of Medicine, Hangzhou, China (SAHZU-2024-031). Six week old female mice (C57BL/6) were purchased from Hangzhou medical college. Sterile titanium plates (diameter, 6 mm) were placed in a 24-well plate. One microliter of MRSA suspension (1 × 10^8^ CFU mL^−1^) in LB was added to each well and cultured at 37 °C for 48 h to form a biofilm on the surface of titanium plates. Mice were anesthetized with aerosolized isoflurane throughout the surgical procedure. The dorsal fur was shaved and the skin was disinfected, followed by fabricating an incision to expose the subcutaneous layer. The biofilm-loaded titanium plate was implanted subcutaneously. One microliter of PCPNAs (200 μg mL^−1^), ε-PLL (200 μg mL^−1^) or normal saline as a control was injected into the infection area at post-treatment day one and two. Six days after different treatments, mice were sacrificed. The collected titanium plate was placed into a 1.5 mL EP tube and immersed in 1 mL of PBS, followed by three cycles of 0.5 min vortex and 10 min sonication. The suspension was diluted 100 times. Subsequently, 100 μL of a diluted suspension was plated on a LB agar plate and then incubated overnight at 37 °C. The number of CFUs was counted and photographed with a camera. Meanwhile, the skin around the implant and main organs (lungs, spleen, heart, liver and kidneys) were harvested and fixed in 4% paraformaldehyde solution. After dehydration with an alcohol gradient, the tissues were embedded in paraffin and 5 μm sections were prepared. The sections were stained with hematoxylin and eosin (H&E) and Wright–Giemsa staining.

### 2.22. Statistical analysis

Each experiment was set to at least three groups in parallel, and all data were expressed as mean ± SD. The Kruskal–Wallis test and Games–Howell test were used for multiple-group comparison. *P* < 0.05 is considered statistically significant. Statistical significance is denoted with ns *p* > 0.05, *p < 0.05, **p < 0.01, and ***p < 0.001. All statistical were analyzed using R software (version 4.3.2) or GraphPad Prism software (Version: 9.5.0).

## 3. Results and discussion

### 3.1. Synthesis and characterization of PCPNAs

**Fig. 1:**
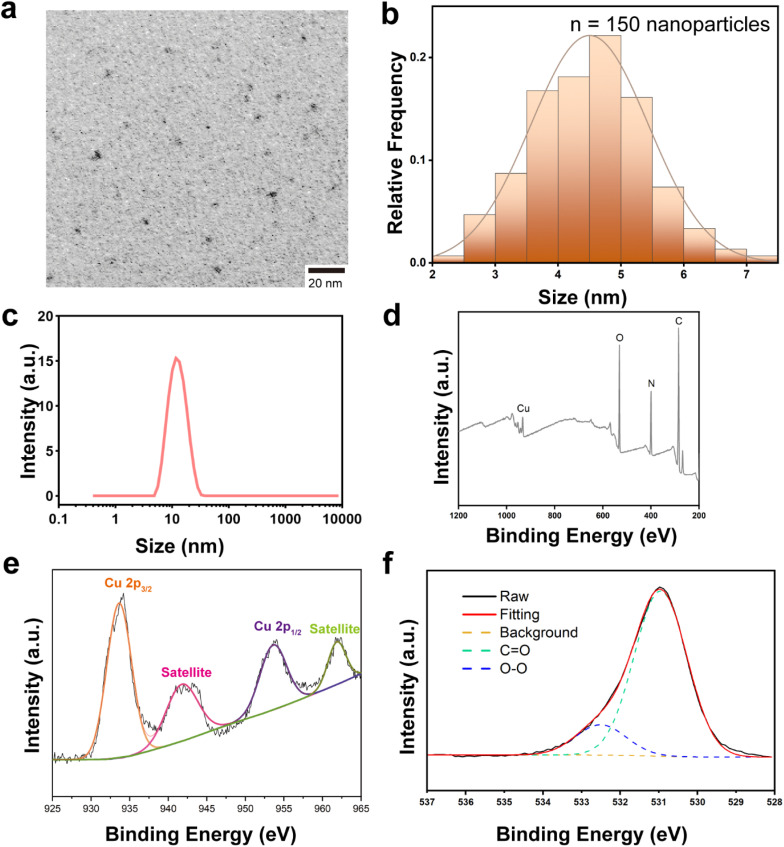
Characterization of PCPNAs. (a) TEM images of PCPNAs. (b) Particle size distribution of PCPNAs at TEM. (c) Dynamic light scattering (DLS) measurement of PCPNAs. (d) XPS spectra of PCPNAs. (e and f) High resolution Cu 2p (e) and O 1s (f) XPS spectra of PCPNAs.

PCPNAs were prepared *via* a novel one-pot synthetic method by the reaction of CuCl_2_, NaOH and H_2_O_2_ in ε-PLL solution for 50 min (Scheme 1). Small sized (∼4.5 nm) ε-PLL-coated CuO_2_ nanodots were observed using TEM (Fig. 1a and b). The nanodots showed good dispensability in water with an average hydrodynamic diameter of ∼13 nm (Fig. 1c) and were highly positively charged, with a zeta potential of +49.67 mV. Furthermore, PCPNAs in PBS (pH 7.4) exhibited a narrow size distribution with a polydispersity index (PDI) of 0.21 and a positively charged surface characterized by a zeta potential of +20.93 mV (Fig. S1†). XPS was conducted to explore the chemical bonds and elemental constituents of the nanodots. The characteristic peaks of C, N, O and Cu were screened in XPS spectra, demonstrating the successful coating of ε-PLL (Fig. 1d). Two main peaks at 933.64 eV and 953.61 eV were accompanied by two satellite peaks at 941.75 and 961.85 eV in high-resolution Cu 2p spectra, suggesting that the main valence state of Cu in the nanodots was +2 (Fig. 1e). In the high-resolution O 1s XPS spectra, two peaks at 530.95 and 532.50 eV were allotted to C=O and O–O, respectively, indicating the presence of ε-PLL and peroxo groups in PCPNAs (Fig. 1f). The content of ε-PLL in PCPNAs was confirmed by ^1^H NMR spectroscopy. As shown in Fig. S2,† the peaks specific to ε-PLL appeared between 1.6 and 3.5 ppm. Peaks at about 1.6 and 1.8 ppm corresponded to β/γ/δ-CH_2_ protons. The peak at 2.3 ppm was assigned to the ε-CH_2_ protons adjacent to the amide bond. The peak at 3.4 ppm corresponded to an α-CH_2_ group adjacent to the protonated α-amino group. FT-IR spectra were conducted to elucidate the molecular interactions within PCPNAs. As shown in Fig. S3 and Table S1,† the adsorption band at 3248 cm^−1^ in PCPNAs (corresponding to the asymmetric stretching vibrations of NH_2_ groups in ε-PLL)^41–43^ exhibited no change, indicating the existence of the primary backbone structure of ε-PLL. The absorption band at 1647 cm^−1^ in raw ε-PLL, assigned to the C=O stretching of amide I,^44,45^ exhibited a shift to 1619 cm^−1^ upon the incorporation into PCPNAs.^46^ Another characteristic absorption band at 1561 cm^−1^ (N–H bending, amide II) was observed in PCPNAs and ε-PLL.^45^ These FTIR spectral alterations collectively confirmed the successful integration of ε-PLL into PCPNAs. As shown in Fig. S4,† the UV absorption spectrum of ε-PLL in an equal-volume mixture of PBS (pH 7.4) and hydrochloric acid (0.1 M) showed that a broad UV absorption peak arises at 201–205 nm (amide bond π → π* transitions), which could be utilized for its quantitative analysis in solution. The UV absorption spectra of PCPNAs were similar to ε-PLL. Based on the standard curve established using ε-PLL (*R*^2^ = 0.995), the mass percentage of ε-PLL in PCPNAs was determined to be 35.02% ± 3.30%, indicating an exceptionally high loading efficiency. To assess the stability of ε-PLL coating in PCPNAs, 50 μg mL^−1^ of the PCPNA solution in PBS (pH 7.4) was sealed in an ultrafiltration tub (10 kDa molecular weight cut-off) for 24 h, followed by centrifugation at 3000×*g* to collect the filtrate. Then, the filtrate was diluted 1 : 1 (v/v) with 0.1 M hydrochloric acid. The amount of ε-PLL coating in the filtrate was quantified using the ε-PLL standard curve method based on UV absorption measurements. The results demonstrated that the concentration of ε-PLL in the filtrate was 7.85 ± 0.38 μg mL^−1^, indicating that over 50% of PCPNAs remained intact. This observation confirmed the robust stability of the ε-PLL coating under the tested conditions.

Collectively, these results demonstrated the successful synthesis of the novel ε-PLL-coated CuO_2_ nanodots.

### 3.2. Fenton-like activity of PCPNAs

**Fig. 2:**
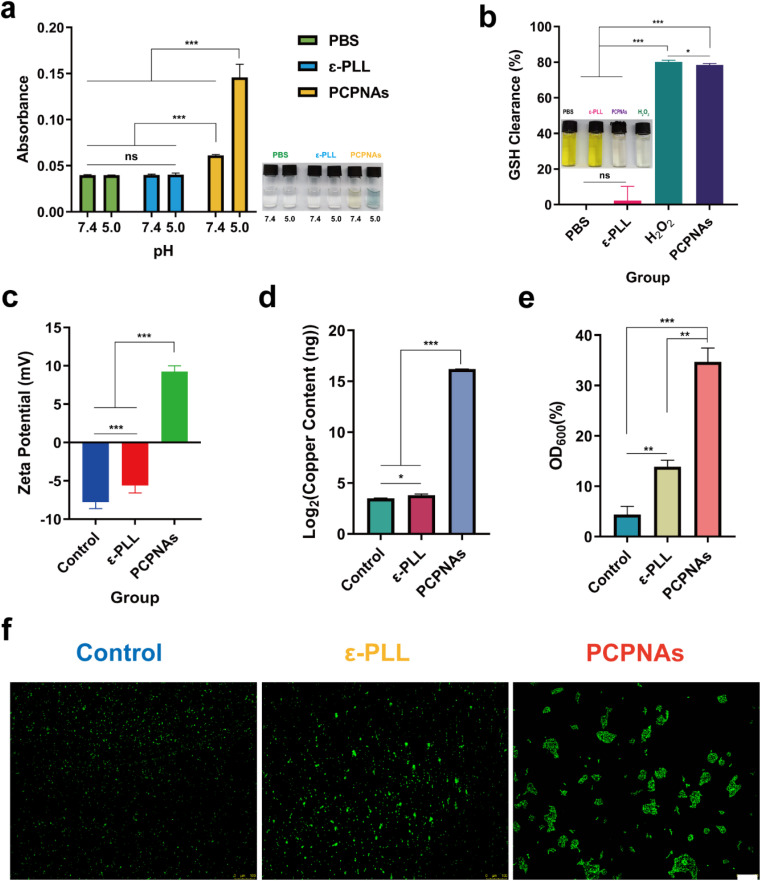
Fenton-like activity and bacterial adhesion characteristic of PCPNAs. (a) TMB-based colorimetric sensing for ˙OH after various treatments at pH 5.0 and 7.4. (b) GSH-depleting properties of various treatments using DTNB. (c) Zeta potential analyses of MRSA exposed to normal saline, ε-PLL and PCPNAs. (d) Copper content of MRSA cells after different treatments. (e) MRSA capture capacity of normal saline, ε-PLL and PCPNAs. (f) Fluorescent images of bacterial aggregation after various treatments (scale bar = 100 μm) (data are shown as mean ± SD, *n* = 3, *p < 0.05, **p < 0.01, ***p < 0.001, ns means *p* > 0.05).

As colorless TMB can be oxidized by ˙OH into blue-green oxidized TMB, a TMB assay was employed to detect ˙OH generated by the Cu^2+^-based Fenton-like reaction between Cu^2+^ and self-supplied H_2_O_2_ of PCPNAs.^47^ As shown in Fig. 2a, the mixed solution of PCPNAs and TMB exhibited a pH-triggered color change to chartreuse under pH 7.4 while it was blue-green at pH 5.0, and its absorbance of 650 nm at pH 7.4 was merely less than half of that at pH 5.0, indicating an acid-induced ˙OH generation characteristic of PCPNAs. In contrast, TMB mixed with ε-PLL or PBS was colorless at different pH values, and its absorbance was lower than the mixed solution of PCPNAs and TMB.

GSH serves as an intracellular antioxidant and can weaken the antibacterial effect of ROS through the consumption of ROS and maintenance of cellular redox homeostasis.^48^ However, a biofilm microenvironment exhibits low pH and abundant GSH.^49^ Thus, damage to the GSH-rich environment is crucial for ROS-based anti-biofilm therapies. In theory, CuO_2_ can decompose to Cu^2+^ and H_2_O_2_ in an acidic environment. GSH further reduces Cu^2+^ to Cu^+^, and is simultaneously oxidized to oxidized glutathione (GSSG). Cu^+^ can catalyze H_2_O_2_ to toxic ˙OH, which is potent oxidant of GSH. As colorless DTNB is able to be reduced by GSH to yield a yellow hybrid disulfide and 2-nitro-5-thiobenzoic acid, Ellman's assay was performed to verify PCPNAs as a potent GSH scavenger.^50^ As shown in Fig. 2b, GSH solution treated with PCPNAs or H_2_O_2_ turned almost colorless, suggesting that PCPNAs or H_2_O_2_ could remarkably deplete GSH. The GSH clearance rate of PCPNAs was as high as 78.51%. Correspondingly, GSH solution mixed with PBS or ε-PLL was yellow.

These results underscored the potential of PCPNAs for pH-responsive and GSH-resistant antimicrobial therapy.

### 3.3. Bacterial adhesion characteristic of PCPNAs

Owing to their positively charged property, PCPNAs can bind to negatively charged bacteria through charge-coupling. The zeta potential of MRSA in normal saline, ε-PLL and PCPNAs treatment groups was −7.78 ± 0.84 mV, −5.61 ± 0.96 mV and 9.24 ± 0.77 mV, respectively. The negative to positive zeta potential reversal of MRSA after treatment with PCPNAs indicated that the bacterial surface charge converted to positive after the adherence of PCPNAs (Fig. 2c). Interestingly, MRSA cells remained negatively charged after ε-PLL treatment, but positively charged after PCPNAs treatment. These results suggested an excellent binding effect and surface charge modification ability of PCPNAs on MRSA cells. The copper content of MRSA cells treated with PCPNAs was much higher than that treated with ε-PLL or normal saline, further suggesting the excellent bacterial adhesion ability of PCPNAs (Fig. 2d). Netting is a practical hunting skill in nature; for example, spiders spin nets to capture their dinner. Like spiders, neutrophils in the human body can release DNA, positively charged histones and other proteins to form net-like NETs to entrap and kill microorganisms.^51^ Positively charged PCPNAs not only possess an adhesion ability, but can also trap bacteria. The OD_600_ values of the supernatant of the bacterial suspension were measured after exposure to different treatments to quantify the bacterial capturing ability of PCPNAs. According to Fig. 2e, the bacterial trapping efficiency of PCPNAs reached 34.69%, far exceeding those of ε-PLL (13.88%) and the control (4.37%). The status of bacteria was detected by fluorescence microscopy to further observe the trapping effect of PCPNAs (Fig. 2f). Evidently, the bacteria exposed to normal saline were highly dispersed. Meanwhile, the bacteria appeared in clusters after PCPNAs and ε-PLL treatment, with larger clusters after PCPNAs. Moreover, bacteria in the PCPNAs group were confined to large separate colonies similar to the model of NETs formation.

These results highlighted the roles of PCPNAs in bacterial adhesion and capture, thereby enhancing ROS-mediated killing effects.

### 3.4. Antibacterial activity *in vitro*

**Fig. 3:**
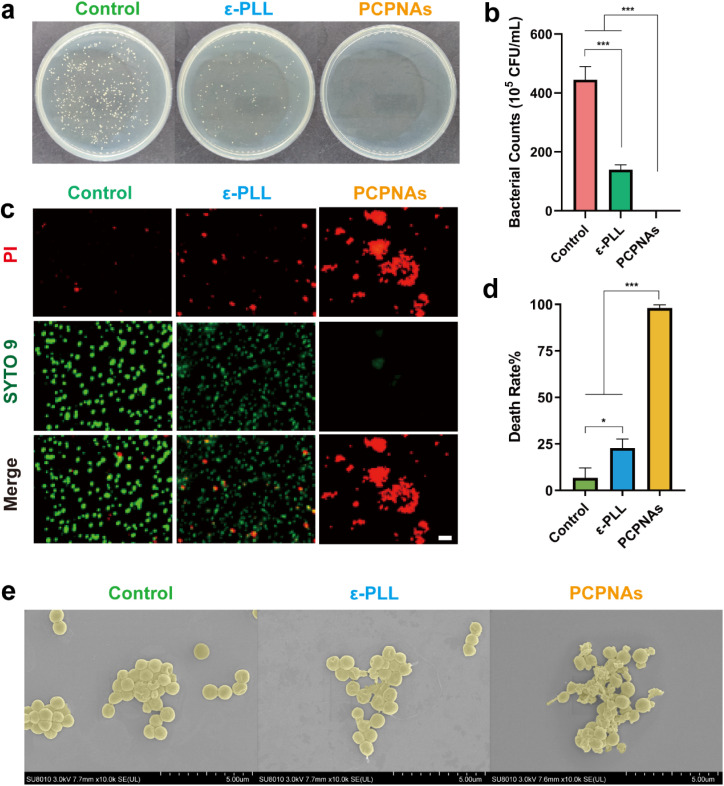
Antibacterial activity *in vitro*. (a) Images of agar plate colonies of MRSA under various treatments. (b) Bacterial density of MRSA exposed to various treatments. (c) Live/dead staining of MRSA (scale bar = 10 μm). (d) Death rate of MRSA after various treatments. (e) SEM images of MRSA treated with normal saline, ε-PLL and PCPNAs (data are shown as mean ± SD, *n* = 3, *p < 0.05, ***p < 0.001).

Plate colony counting was performed to evaluate the antibacterial activity of PCPNAs. The results in Fig. 3a and b show that both ε-PLL and PCPNAs exhibited antibacterial effects. However, ε-PLL (100 μg mL^−1^) only showed a certain inhibitory effect on bacterial growth, as the bacterial density of MRSA treated with ε-PLL still reached up to 139.5 ± 16.82 × 10^5^ cfu mL^−1^, compared with 445.3 ± 44.4 × 10^5^ cfu mL^−1^ for the control group. On the basis of these results, ε-PLL was not sufficient to completely eliminate MRSA. It is noteworthy that PCPNAs displayed prominent antibacterial performance. PCPNAs at 100 μg mL^−1^ achieved complete sterilization of MRSA, demonstrating a 7.65 log_10_ CFU reduction (*p* < 0.001) compared with the untreated controls, which is equivalent to eliminating 99.99998% of bacterial populations. The results showed that PCPNAs demonstrated vastly superior antibacterial efficacy compared to ε-PLL alone against MRSA (5 × 10^8^ CFU mL^−1^) at matched concentrations (100 μg mL^−1^).

As shown in Fig. S5,† PCPNAs exhibited robust concentration-dependent antimicrobial activity against MRSA, with a relatively low MIC value of 100 μg mL^−1^, implying that PCPNAs can effectively inhibit the growth of MRSA at a moderate concentration and have potential as a viable alternative to traditional antibiotics for treating MRSA-related infections. The time-kill kinetics assay further elaborated on the bactericidal mechanism of PCPNAs. The rapid 1.86 log_10_ CFU mL^−1^ reduction within 4 h at 2 × MIC (200 μg mL^−1^) showcases the fast-acting nature of PCPNAs. By 8 h, the detection limit was reached (<10 CFU mL^−1^) and the persistent suppression lasted until 24 h, showing that PCPNAs rapidly only killed MRSA and prevented their regrowth over an extended period. This long-term suppression effect is valuable in preventing the recurrence of implant-associated infections.

To further confirm the notable therapeutic effect of PCPNAs, a live/dead staining assay was conducted. SYTO 9 dye can label live and dead bacteria, which permeates into both integrated and damaged prokaryotic cell membranes and exhibits enhanced green fluorescence upon binding to bacterial nucleic acids.^52^ PI can only penetrate damaged cell membranes and embed nucleic acids to generate red fluorescence, and its insertion can cause a decrease in SYTO 9 staining fluorescence.^53^ As displayed in Fig. 3c, bacteria in the control group were highly dispersed and emitted strong green fluorescence. Red fluorescence was enhanced and the bacteria were aggregated in a small scale after ε-PLL treatment compared with the control group, indicating that some bacteria had been inactivated. Some beige to orange fluorescence was detected in the ε-PLL group, suggesting that some bacterial membranes were slightly damaged, allowing some of the PI to enter bacteria and embed nucleic acids but it was not high enough to supplant SYTO 9 binding to nucleic acids, which causes beige to orange fluorescence. These results demonstrated that ε-PLL alone had insufficient antibacterial ability. In contrast, PCPNAs displayed excellent antibacterial activity; there was an extensive distribution of red fluorescence and only faint green fluorescence. The overall trend of the death rate was in line with the results from the plate colony counting examination (Fig. 3d).

The morphology changes of MRSA in different groups were further detected by SEM (Fig. 3e). In the control group, MRSA preserved normal morphology with no membrane damage. As a natural antibacterial cationic peptide, ε-PLL can interact with negatively charged bacteria to disturb cell membrane integrity.^54^ Some of the bacteria with abnormal shapes and ruptured membranes were observed in the ε-PLL group. In contrast, a large quantity of bacteria in the PCPNA group was severely damaged, with irregular morphologies and lacerated membranes, further confirming the strongest antibacterial effect of PCPNAs.

Taken together, these results established PCPNAs as a promising platform for combating drug-resistant infections by integrating electrostatic adhesion, ROS-mediated killing, and long-term bacterial suppression.

### 3.5. Antibiofilm activity

**Fig. 4:**
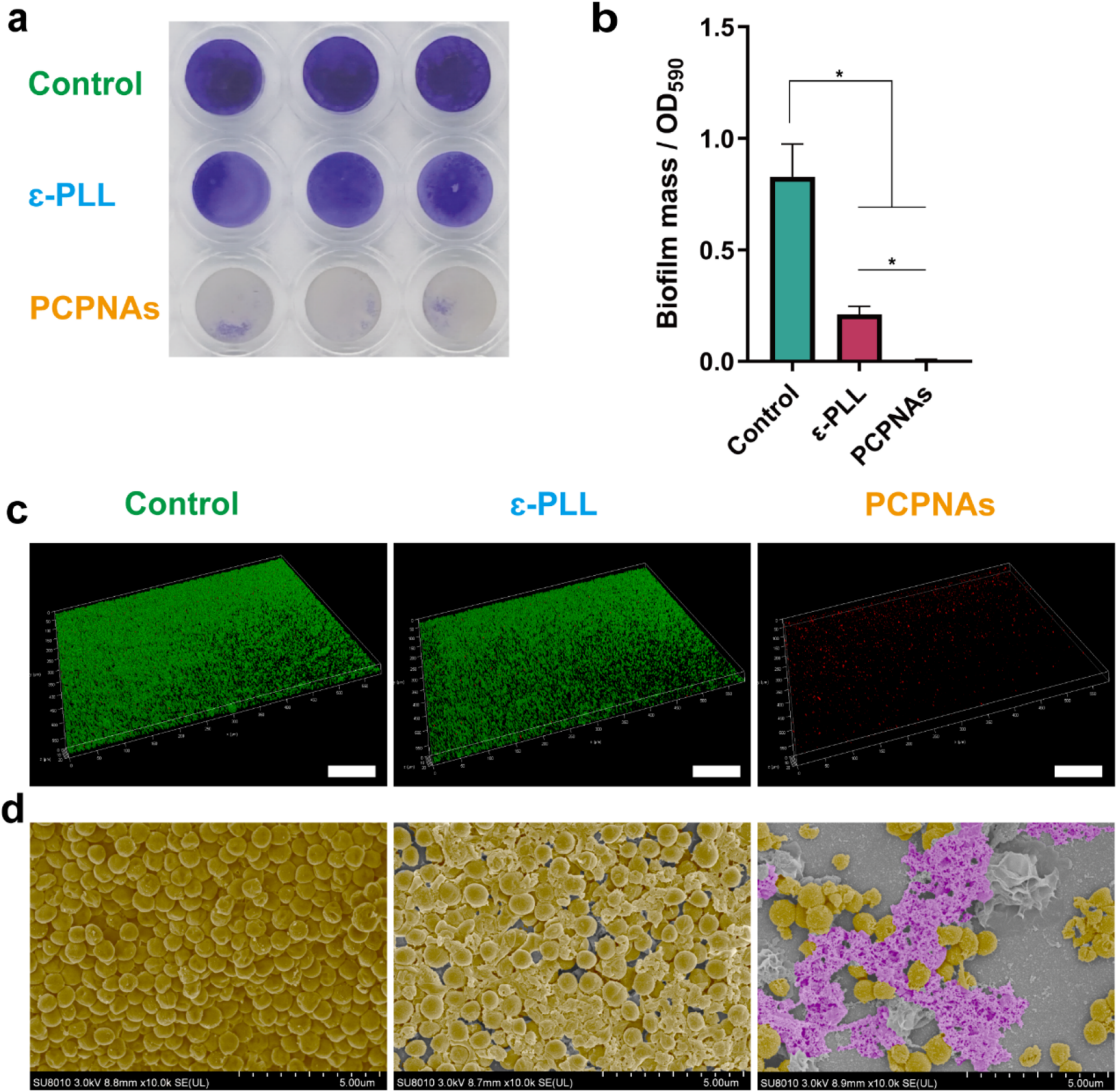
Biofilm dispersion assays. (a) Crystal violet staining of biofilms. (b) Biofilm mass of MRSA. (c) 3D CLSM images of MRSA biofilms treated with normal saline, ε-PLL and PCPNAs (red fluorescence: MRSA biofilms stained with PI; green fluorescence: Calcein-AM; scale bar = 100 μm). (d) False-colored SEM images of MRSA biofilms (the pink regions represent PCPNAs and the golden areas correspond to MRSA biofilms) (data are shown as mean ± SD, *n* = 3, *p < 0.05).

Bacterial biofilms organized by microbial communities are totally different from free-living bacterial cells, exhibiting an enhanced resistance to antibiotics.^55^ Eliminating biofilms is the key to clear bacterial infections. A Crystal violet assay was performed to assess the antibiofilm activity of PCPNAs against the mature biofilm of MRSA and the biofilm mass was quantified using the corresponding absorbance. As shown in Fig. 4a, relatively intact and partially reduced biofilms were observed in control and ε-PLL groups, respectively, whereas the biofilms in the PCPNAs group were nearly completely eradicated. PCPNAs mediated 99.58% biofilm eradication, showing statistically complete clearance (*p* < 0.05 *vs.* 0% in the control group). Meanwhile, the results of the biofilm mass quantification were in consistent with the general observation (Fig. 4b). The control and PCPNAs groups displayed the highest and lowest absorbance, respectively. These results showed the robust biofilm dispersion ability of PCPNAs.

The biofilm dispersion ability of PCPNAs was further observed using 3D confocal laser scanning microscope (CLSM). As shown in Fig. 4c, a very weak PI fluorescence signal was detected on the biofilm after treatment with PCPNAs. The biofilm in this group appeared the thinnest and sparsest, suggesting that PCPNAs have a robust biofilm eradication effect. In contrast, biofilms in the control and ε-PLL groups showed dense Calcein-AM fluorescence signals, indicating that massive viable biofilms remained in these groups.

In addition, the antibiofilm ability of PCPNAs was investigated by SEM. As shown in Fig. 4d, integrated and dense biofilm embedded with a plump spherical shape of bacteria was observed in the control group. The ε-PLL group showed a loose biofilm with some lacerated bacterial cells, indicating that the biofilm could not be completely disrupted under this condition. In contrast, the biofilm treated with PCPNAs was highly dispersed and broken bacterial cells were observed, suggesting that PCPNAs could effectively destroy the biofilm.

The advancement of innovative antibacterial methodologies has progressively transcended conventional antibiotic frameworks in contemporary scientific exploration. Cutting-edge methodologies, particularly self-organizing nanocomposites, exhibit synergistic antibacterial mechanisms through programmable structural dynamics and coordinated functional cooperation.^56–58^ More interestingly, as illustrated in Fig. S6,† similarity to NETs preying on microorganisms, PCPNAs could agglomerate together to form larger interconnected webs to capture and encircle the biofilm bacteria, demonstrating a vigorous bacterial entrapment ability of PCPNAs. The targeted self-assembly of PCPNAs into bactericidal web-like structures was a pathogen-selective process orchestrated by the unique physicochemical interplay between PCPNAs and biofilm components. Negatively charged EPS components, such as bacterial DNA and polysaccharides, weakened the surface charge of PCPNAs through charge neutralization, while acting as biological templates to induce their orientational aggregation at the biofilm periphery. This process ultimately led to the assembly of 3D aggregates with web-like structures. The resulting web-like structures physically immobilized MRSA cells, while localized ROS generation from CuO_2_ decomposition ensured biofilm eradication. This biofilm-microenvironment-mediated nanostructural remodeling replicated natural NETs through a dual-layered defense algorithm: biochemical pattern recognition (pathogen-derived EPS provides molecular fingerprints guiding nanoparticle localization) and stimuli-interpreted activation (acidic biofilm infection microenvironment converted dormant CuO_2_ into oxidative bactericidal agents *via* the Fenton-like reaction). This condition-responsive therapeutic delivery system operates on immunological “detect-and-act” mechanisms and achieves spatiotemporally restricted activity at the MRSA biofilm infection site.

### 3.6. Antibacterial mechanism of PCPNAs

**Fig. 5:**
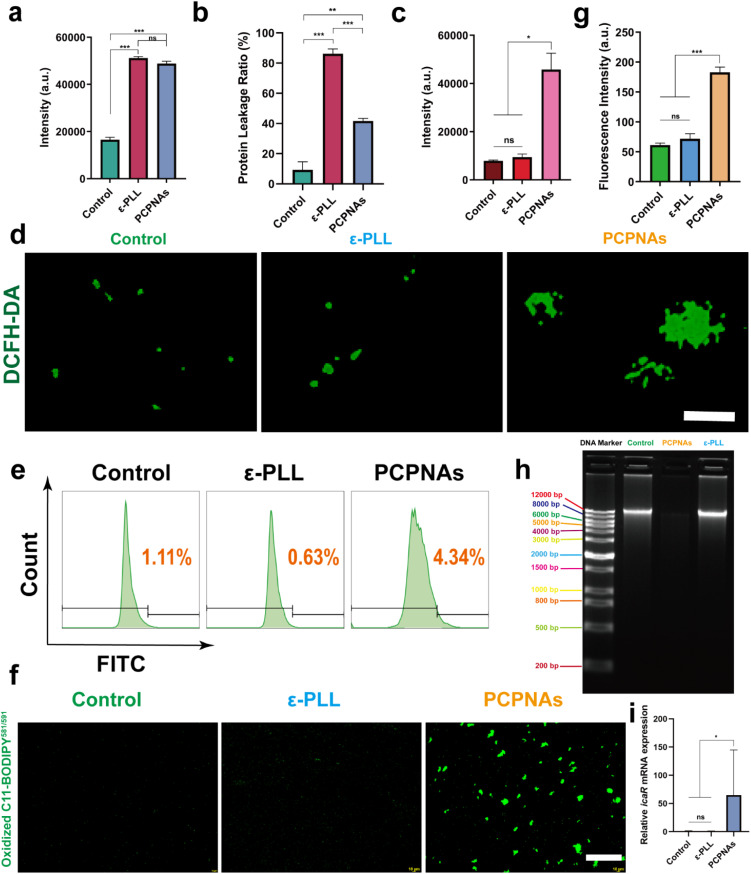
Antibacterial mechanism of PCPNAs. (a) Membrane depolarization of MRSA under different treatments using DiSC35. (b) Protein leakage rate from MRSA after various treatments. (c) Intracellular ROS production of MRSA exposed to various treatments using DCFH-DA. (d) Fluorescent images of ROS in MRSA under various treatments (scale bar = 10 μm). (e) Representative results of flow cytometry of lipid peroxidation in MRSA exposed to normal saline, ε-PLL and PCPNAs. (f) Probe-labeled green fluorescence of oxidized C11-BODIPY581/591 images of MRSA after different treatments (scale bar = 100 μm). (g) Semiquantitative analysis of green fluorescence intensity of oxidized C11-BODIPY581/591 in MRSA under different treatment conditions. (h) Agarose gel electrophoresis result of MRSA gDNA under different treatments. (i) Effect of PCPNAs treatment on biofilm-related icaR gene expression (data are shown as mean ± SD, *n* = 3, *p < 0.05, **p < 0.01, ***p < 0.001, ns means *p* > 0.05).

To clarify the antibacterial mechanism of PCPNAs, the cytoplasmic membrane depolarization assay was first performed. The transmembrane potential maintaining multiple functions is vital to the survival of bacteria.^59^ Dissipating transmembrane potential causes leakage of cell contents and bacterial death.^60^ DiSC_3_5 is a fluorescent probe. The dye accumulates in cells on polarized membranes resulting in fluorescence self-quenching, while it is released upon membrane depolarization, provoking fluorescence dequenching.^61^ As anticipated, treatment of MRSA with PCPNAs and ε-PLL augmented DiSC_3_5 fluorescence, indicating that PCPNAs and ε-PLL targeted and interacted with bacterial membranes and caused membrane depolarization (Fig. 5a).

The leaking of intracellular protein ascribed to the changed bacterial cytoplasmic membrane permeability was detected using Coomassie brilliant blue G-250. It was found that both PCPNAs and ε-PLL increased cytoplasmic leakage, suggesting disrupted membrane permeability after PCPNAs or ε-PLL treatment (Fig. 5b). Interestingly, the protein leakage ratio in the PCPNAs group was lower than the ε-PLL group. It is possibly because positively charged PCPNAs on the bacterial membrane acted as a physical barrier against the spread of harmful proteins.

Intracellular ROS production of bacteria was detected *via* a fluorescent ROS probe (DCFH-DA) to further explore the sterilization mechanism of PCPNAs. As shown in Fig. 5c, there was no difference in ROS yield between the ε-PLL and control groups. In contrast, there was an upsurge in ROS generation in response to PCPNAs, indicating that Fenton-like reaction-based PCPNAs could induce an intracellular ROS burst in MRSA. Fluorescence images showed faint green fluorescence in ε-PLL and control groups, while there was high intensity aggregated green fluorescence signals in the PCPNAs group, further confirming the substantial production of ROS (Fig. 5d).

The LPO level in MRSA was further evaluated. An LPO-specific fluorescent probe (C11-BODIPY^581/591^) was employed to characterize bacterial membrane lipid peroxides. The maximum emission peak of C11-BODIPY^581/591^ is blue shifted from 595 nm (red) to 520 nm (green) when it is oxidized.^62^ Flow cytometry demonstrated an increased LPO accumulation in bacteria post PCPNAs treatment (Fig. 5e). Fluorescence images in Fig. 5f showed that both ε-PLL and control groups exhibited dim green fluorescence, while enhanced green fluorescence was observed in the PCPNAs group. Semiquantitative analyses of the fluorescence images supported that PCPNAs treatment increased the LPO level (Fig. 5g).

To further explore the biofilm dispersion mechanism of PCPNAs, DNA gel electrophoresis was performed. Bacteria within the biofilm are encased in and protected by extracellular polysaccharides (EPSs) of the biofilm.^63^ Extracellular DNA (eDNA) is a primary composition of EPS, which can stabilize charges and offer structural rigidity of the biofilm matrix.^64^ As eDNA is similar to intact genomic DNA (gDNA),^65^ the gDNA was extracted and used to test the eDNA destructive capacity of PCPNAs. Agarose gel electrophoresis revealed that the DNA bands of both ε-PLL and control groups were concentrated at 12 000 bp, implying the integrity of gDNA (Fig. 5h). Meanwhile, no obvious DNA band was observed in the PCPNAs group, indicating that PCPNAs completely fragmented the gDNA.

In addition, the transcript levels of the biofilm-related gene *icaR* was determined using qRT-PCR to further clarify the biofilm dispersion mechanism of PCPNAs. The *icaR* gene is a member of the TetR family and is a negative regulator of biofilm formation of *Staphylococcus aureus*.^66^ Wang *et al.*^67^ showed that *Ginkgo biloba* exocarp extracts can inhibit the MRSA biofilm-forming ability by up-regulating *icaR* gene expression levels. Rao *et al.*^68^ synthesized a small-molecule compound SYG-180-2-2 that can inhibit the formation of MRSA biofilms by up-regulating the expression of the *icaR* gene. Our results showed that the expression of *icaR* was up-regulated after PCPNAs treatment compared to both ε-PLL and control groups, suggesting the Inhibitive effect of PCPNAs on MRSA biofilms (Fig. 5i).

PCPNAs exhibited dual bactericidal and antibiofilm activities through synergistic mechanisms, including membrane potential disruption, ROS-mediated oxidative stress, lipid peroxidation, and transcriptional modulation of a critical biofilm-related gene. These results positioned PCPNAs as a promising therapeutic candidate for addressing drug-resistant infections.

### 3.7. Cytotoxicity of PCPNAs

**Fig. 6:**
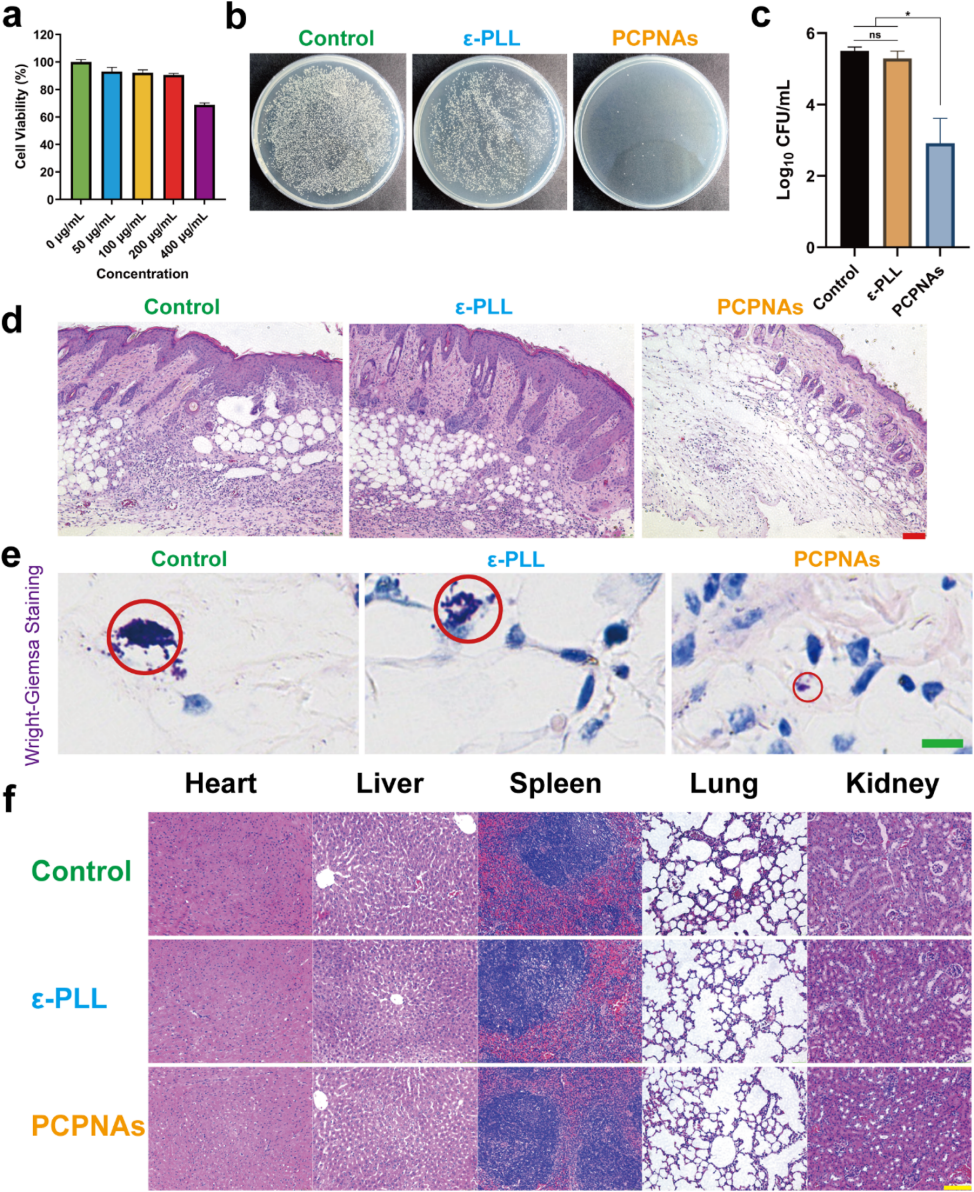
Biocompatibility and *in vivo* antibacterial activity of PCPNAs. (a) Relative viability of C2C12 cells cultured with PCPNAs at gradient concentrations. (b) Photographs of agar plate colonies of MRSA in titanium plates receiving different treatments *in vivo* (*n* = 4). (c) CFU counts corresponding to agar plate colonies of MRSA (data are shown as mean ± SD, *n* = 4, *p < 0.05, ns means *p* > 0.05). (d) H&E staining of the peri-implant tissues after various treatments (*n* = 4, scale bar = 100 μm). (e) Wright–Giemsa staining of the peri-implant tissues after various treatments (*n* = 4, red circle refers to the substantial bacterial infiltration, scale bar = 10 μm). (f) H&E staining images of the major organs exposed to various treatments after six days (*n* = 4, scale bar = 100 μm).

Biocompatibility of antibacterial nanomaterial is one of the principal requirements for its clinical application. Thus, we performed CCK-8 tests to assess the cytotoxicity of PCPNAs as an indicator of biocompatibility. To better reflect the biosafety of PCPNAs, C2C12 cells were exposed to PCPNAs at different concentrations for 24 h. As shown in Fig. 6a, cell viabilities of C2C12 treated with PCPNAs at 50, 100 and 200 μg mL^−1^ were maintained at more than 80%, while the viability of C2C12 cells began to decline at concentrations above 200 μg mL^−1^, with a relative survival rate below 70%, confirming dose-dependent cytotoxicity of PCPNAs. Notably, the viability of cells was maintained higher than 90% at 200 μg mL^−1^ PCPNAs, suggesting a low cytotoxicity of PCPNAs. Based on these results, the concentration of PCPNAs applied *in vivo* antibacterial activities was set as 200 μg mL^−1^.

The results showed that PCPNAs presented a promising solution for addressing implant-associated infections by combining bactericidal efficacy and biocompatibility.

### 3.8. *In vivo* antibacterial activity of PCPNAs

Encouraged by the excellent antibacterial performance of PCPNAs *in vitro*, we applied it *in vivo* for the treatment of implant-associated infection. A MRSA-contaminated titanium implant was placed subcutaneously to build a mouse implant-related infection model, and the antibacterial activity of PCPNAs was further investigated. To clarify the antibacterial property of PCPNAs *in vivo*, the titanium implants were harvested on day 6 and plate colony counting was performed to quantify the antibacterial effect. As shown in Fig. 6b, abundant bacterial colonies were observed in the control group and there was a minor reduction in the quantity of bacterial colonies in the ε-PLL group after subcutaneous implantation for 6 days. In comparison, the number of colonies in the PCPNA group was significantly lower (*p* < 0.05) than the other two groups. Quantitative statistics of the bacterial colonies showed that the bacterial densities of the control and ε-PLL groups was 5.50 ± 0.11 log_10_ CFU mL^−1^ and 5.29 ± 0.21 log_10_ CFU mL^−1^, respectively, while the bacterial concentration in the PCPNAs group intensively declined to 2.92 ± 0.70 log_10_ CFU mL^−1^, which was significantly lower than the other groups (Fig. 6c). Based on the above results, PCPNAs displayed an ideal anti-bacterium effect *in vivo*.

Persistent bacterial infection is inclined to induce excessive inflammation, resulting in chronic or systemic inflammatory diseases.^69,70^ Therefore, H&E staining was conducted on the peri-implant tissues to evaluate the inflammatory response of the tissues surrounding the implant. As shown in Fig. 6d, after implantation for 6 days, a large amount of inflammatory neutrophils was recruited to the subcutaneous tissue in control group, implying that an excessive inflammatory response to bacterial infection was activated. A considerable number of inflammatory cells infiltrated to the peri-implant tissues in the ε-PLL group, indicating insufficient clearance of infection after ε-PLL treatment. In contrast, a few inflammatory cells were scattered in the peri-implant tissues in the PCPNAs group, demonstrating that the inflammation was reduced and the infection was controlled compared with the other two groups. Furthermore, Wright–Giemsa staining was performed to assess the infection burden on the peri-implant tissues. As shown in Fig. 6e, the Wright–Giemsa staining of the control and ε-PLL groups showed that a certain number of bacterial colonies existed in the peri-implant tissues, indicating that the bacterial infection was serious in these groups. In contrast, the PCPNAs group had fewer bacteria, suggesting that the bacterial infection was less than those in the control and ε-PLL groups.

To investigate the biocompatibility of PCPNAs *in vivo*, the five major organs were harvested on day 6 after implantation, including the heart, liver, spleen, lungs, and kidneys, and stained by H&E in three groups (Fig. 6f). H&E staining examination of these organs displayed negligible histological changes after the administration of PCPNAs compared with the other groups. No evidence of necrosis, atrophy, fibrosis, hemorrhage, hyperplasia, inflammation or other pathological changes were observed for the five major organs in the PCPNAs group. Taken together, PCPNAs demonstrated *in vivo* efficacy against implant-associated MRSA infection by reducing bacterial load and inflammation *via* electrostatic targeting and ROS generation while maintaining biocompatibility, suggesting its suitability for clinical applications.

## 4. Conclusions

We successfully proposed a novel ε-PLL coated CuO_2_ nanoplatform, denoted as PCPNAs, to constitute biomimetic nano-NETs for combating implant-associated infections from methicillin-resistant *Staphylococcus aureus*. The cationic character enabled PCPNAs to anchor on the surface of the pathogenic bacteria and trap them. The nano-sized CuO_2_ NPs exhibited excellent Fenton-like performance, including robust ROS generation as well as GSH scavenging ability. *In vitro* experiments illustrated that PCPNAs had satisfactory antibacterial properties against drug-resistant bacteria and efficient clearance of mature biofilms through Fenton-like reaction-based chemodynamic therapy. The exploration of the potential antibacterial mechanisms showed that PCPNAs caused bacterial cytoplasmic membrane depolarization and enhanced bacterial cytoplasmic membrane permeability and intracellular ROS and LPO generation as well as DNA damage. More interestingly, PCPNAs could self-assemble into web-like structures to trap and kill biofilm bacteria. In addition, PCPNAs showed low cytotoxicity and satisfactory biocompatibility *in vivo* and displayed ideal antibacterial and anti-inflammatory effects in a mouse model of implant-associated infection. Taken together, the biomimetic nano-NET strategy based on PCPNAs exhibits excellent antibacterial activity and provides a new therapeutic option for the treatment of MRSA-related implant-associated infection.

## Data availability

All the data generated and/or analysed during this study are included in the article, and any further inquiries can be directed to the corresponding author.

## Author contributions

Conceptualization, H. T. and D. H.; data curation, H. X.; formal analysis, H. X.; investigation, H. T.; methodology, H. X.; project administration, H. T. and D. H.; resources, H. T.; supervision, D. H.; validation, H. T.; visualization, H. X.; writing—original draft, H. X.; writing—review and editing, H. T. and D. H. All authors have read and agreed to the published version of the manuscript.

## Conflicts of interest

The authors declare no conflicts of interest.

## Supplementary Material

RA-015-D5RA00367A-s001
