# Supplementary material for: A biomimetic nano-NET strategy for the treatment of MRSA-related implant-associated infection
Source: RSC Adv. 2025 May 7;15(19):14821–37. doi: 10.1039/d5ra00367a (PMC12057620; doi:10.1039/d5ra00367a)
Supplement: RA-015-D5RA00367A-s001 [file RA-015-D5RA00367A-s001.pdf]

Supplementary Information (SI) for RSC Advances.
This journal is © The Royal Society of Chemistry 2025

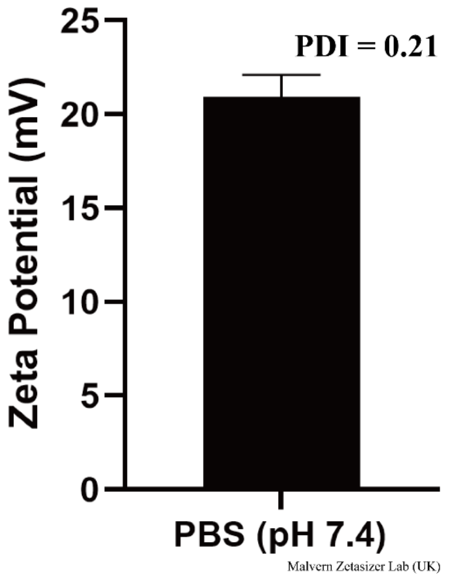

Figure S1 The PDI and zeta potential of PCPNAs in PBS.

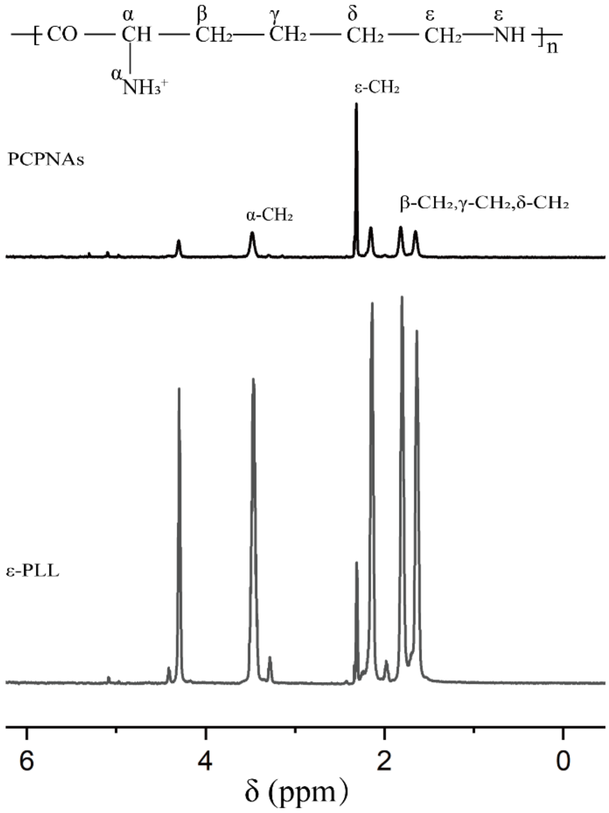

Figure S2 ^{1}H-NMR spectrum of PCPNAs and ε-PLL.

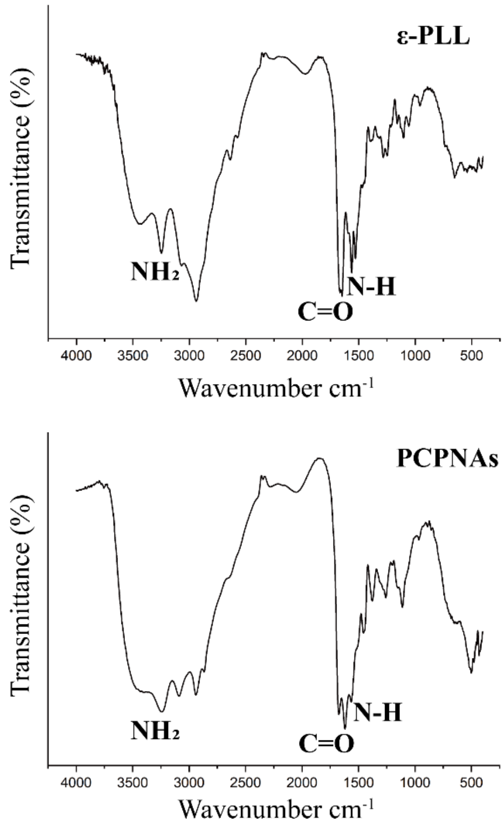

Figure S3 FT-IR spectra of ε-PLL and PCPNAs between 400 and 4000 cm^{-1}.

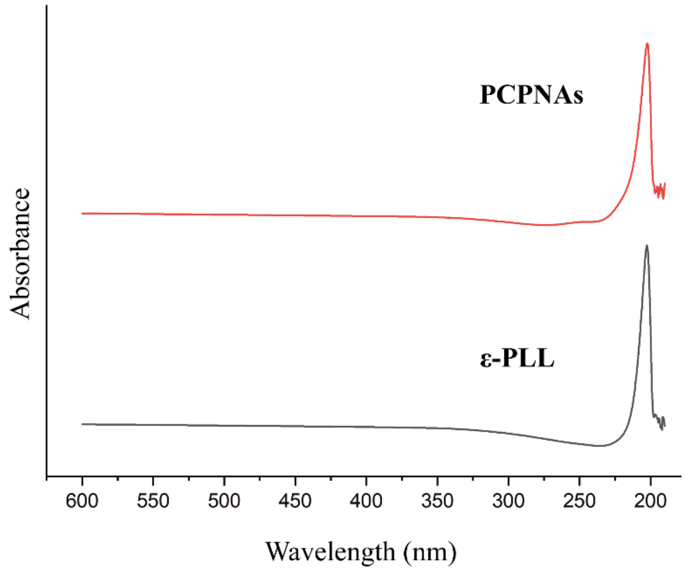

Figure S4 UV absorption spectra of PCPNAs and ε-PLL.

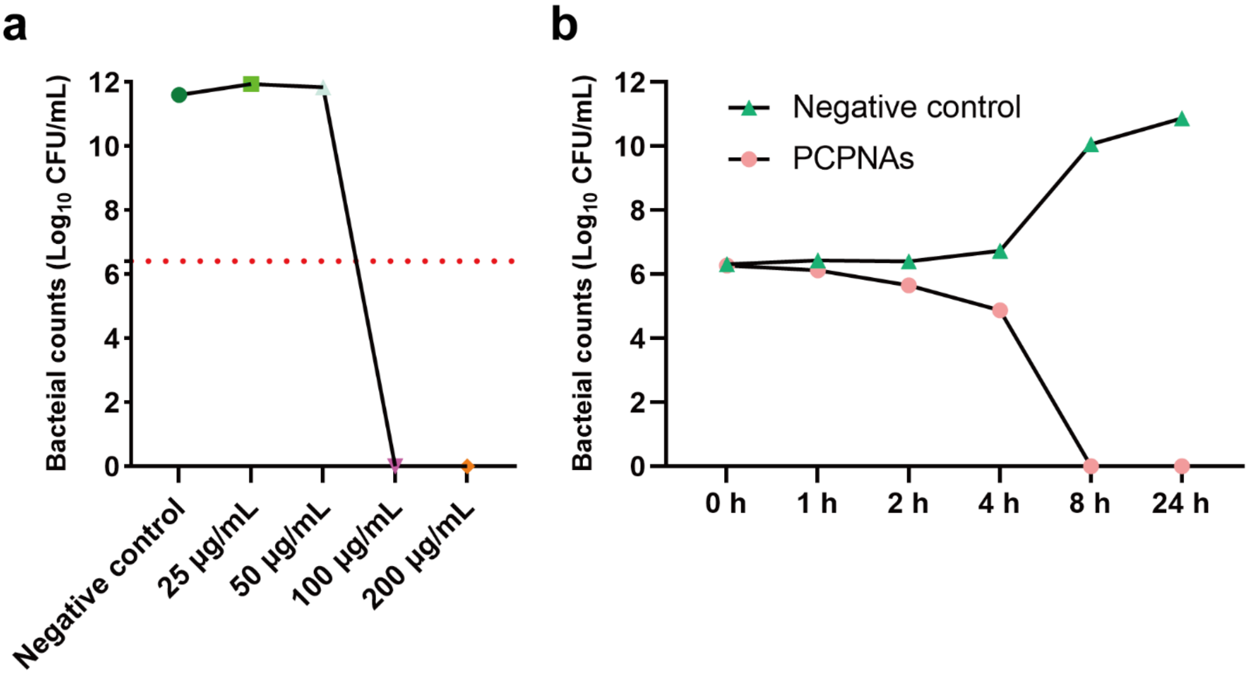

Figure S5 a) The MIC of PCPNAs (the red line indicates the initial bacterial counts). b) The time-kill kinetics assay of PCPNAs.

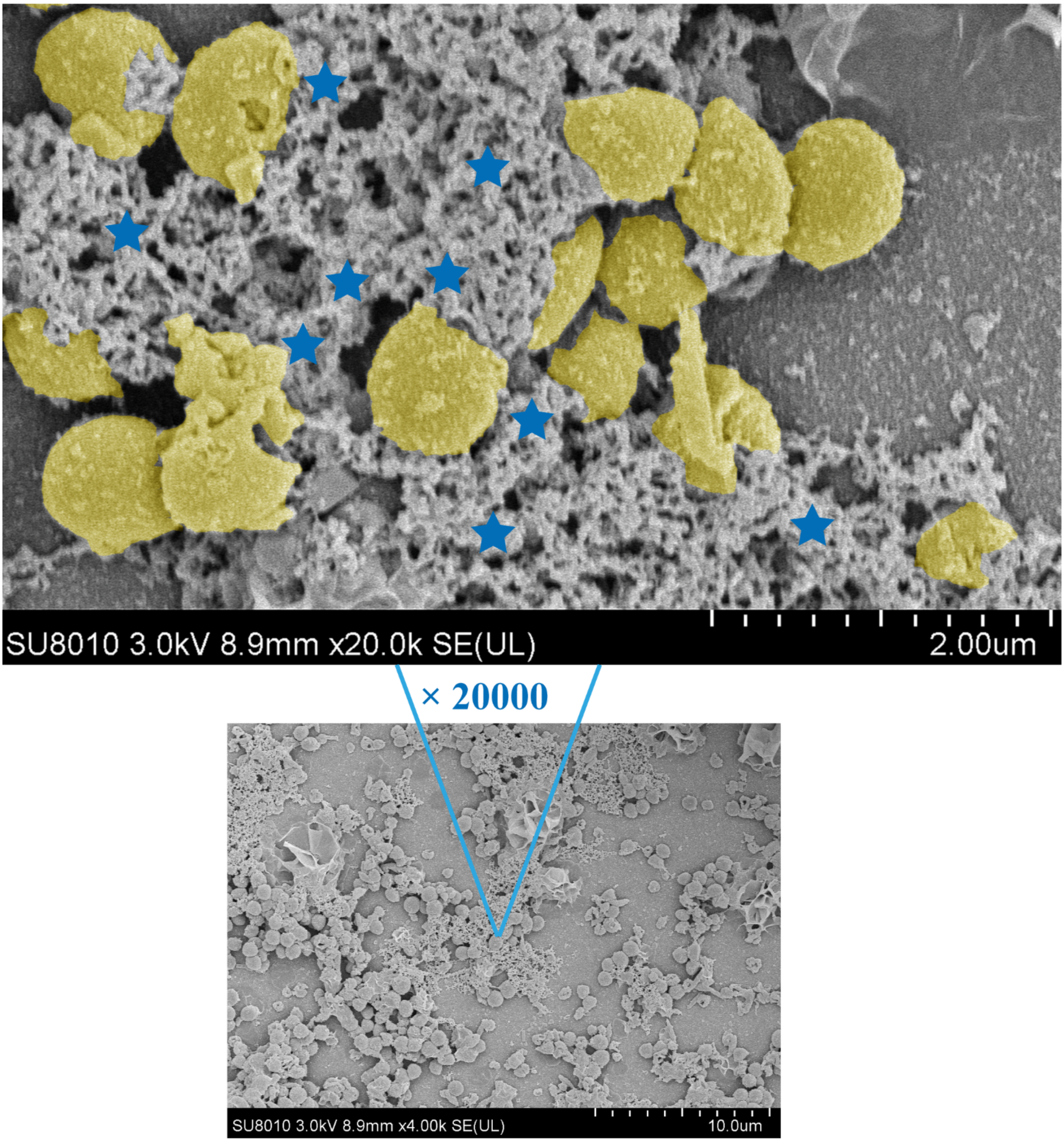

Figure S6 False-colored SEM images of web-like structures of PCPNAs at MRSA biofilms (the blue stars indicated the porous network structure of PCPNAs and the golden areas correspond to MRSA cells).

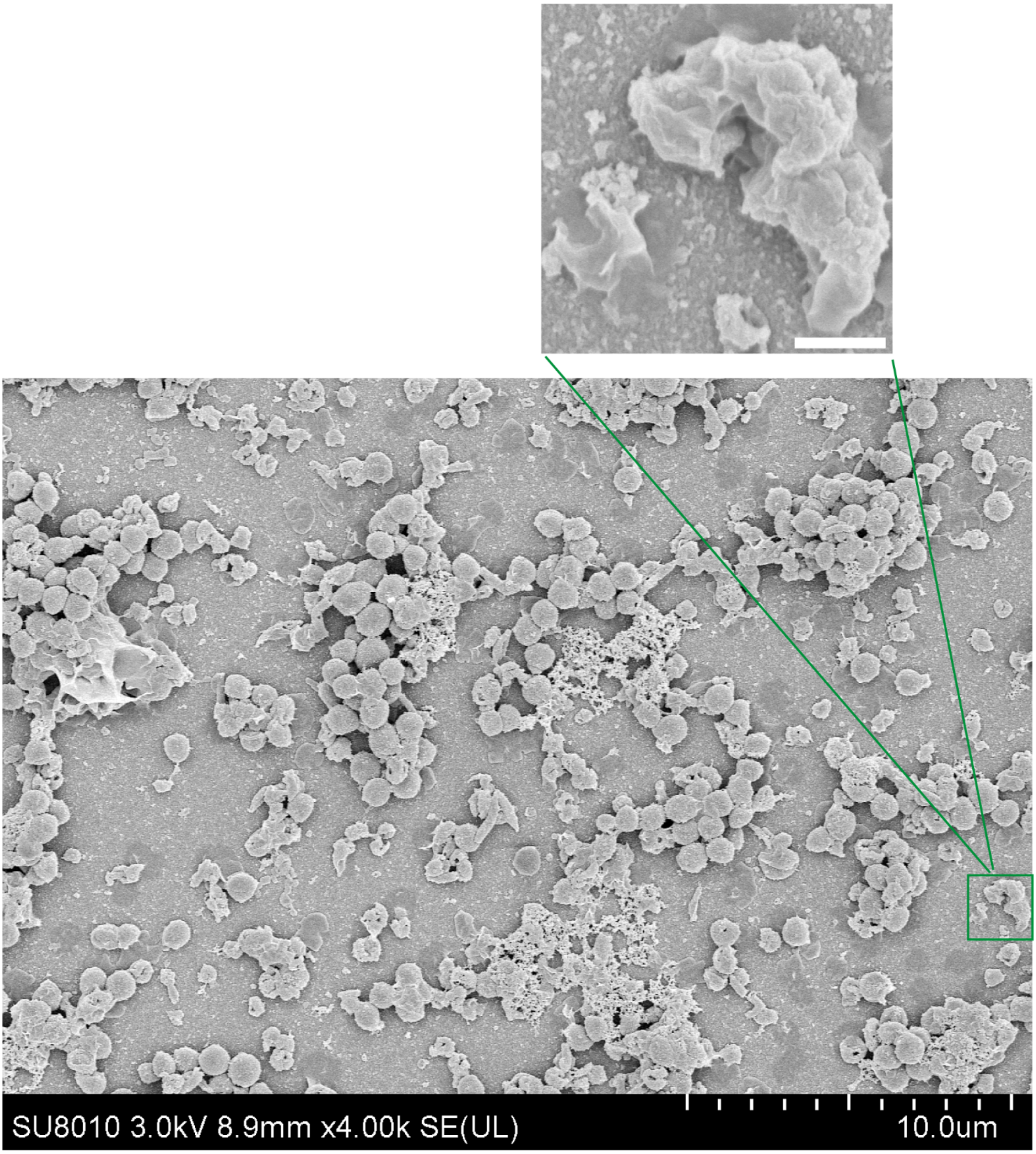

Figure S7 High-resolution SEM images of biofilm disruption by PCPNAs (Scale bar = 0.5 μm).

Table S1. FT-IR Peak Assignments for ε-PLL and PCPNAs

Wavenumber (cm^{-1})	Assignment	References
**3248**	NH_2 asymmetric stretching	[41-43]
**1647 (raw ε-PLL)**	C=O stretching	[44, 45]
**1619 (PCPNAs)**	Shift C=O stretching	[46]
**1561**	N–H bending	[45]
